# Enhancement in Tomato Yield and Quality Using Biochar Amendments in Greenhouse under Salinity and Drought Stress

**DOI:** 10.3390/plants13121634

**Published:** 2024-06-13

**Authors:** Abdullah Obadi, Abdulaziz Alharbi, Abdulrasoul Alomran, Abdulaziz G. Alghamdi, Ibrahim Louki, Arafat Alkhasha, Thabit Alqardaeai

**Affiliations:** 1 Plant Production Department, King Saud University, Riyadh 11451, Saudi Arabia; obadi201@gmail.com (A.O.); akharbi@ksu.edu.sa (A.A.); thabit.alqardaeai@ksu.edu.sa (T.A.); 2 Soil Science Department, King Saud University, Riyadh 11451, Saudi Arabia; agghamdi@ksu.edu.sa (A.G.A.); ibrahim.loukimi@hotmail.com (I.L.); aalkhasha@ksu.edu.sa (A.A.)

**Keywords:** biochar, fruit, irrigation, mineral content, plant, quality, saline water, soil, tomato, yield

## Abstract

Enhancing saline water productivity in arid regions is essential for sustainable agriculture. Adding biochar can improve the quantity and quality of tomato yield under higher levels of salinity and lower levels of irrigation. The experiment aimed to evaluate the effects of biochar on enhancing tomato fruit quality and yield under salinity and drought stress. The experiment combines two treatments for irrigation water quality (0.9 and 2.3 dS m^−1^), four irrigation levels (40, 60, 80, and 100%) of crop evapotranspiration (ETc), and the addition of 5% of biochar to treated soil (BC_5%_) and untreated soil (BC_0%_). The results showed that the decrease in the water quality and irrigation levels negatively impacted the yield and properties of tomato fruit, while 5% of biochar application positively improved the yield. Adding biochar decreased the tomato yield by 29.33% and 42.51% under lower-saline-irrigation water than the control, negatively affecting the fruit’s physical parameters and mineral content. In contrast, adding biochar and irrigating with saline water at 60% of ETc improved the firmness and quality characteristics of the fruit by 56.60%, 67.19, 99.75, and 73.57% for vitamin C (VC), total titratable acidity (TA), total soluble solids (TSS), and total sugars (TS), respectively, compared to the control, and also reduced the sodium content of the fruits under all irrigation levels compared to untreated plants by biochar. Generally, biochar with saline water under deficit irrigation with 80 and 60% of ETc could be an excellent strategy to enhance the qualitative characteristics of tomato fruits and save approximately 20–40% of the applied water.

## 1. Introduction

The tomato plant (*Solanum lycopersicum* L.) is widely recognized as one of the most popular and consumed vegetables globally [1]. Due to the rising awareness regarding the advantages of healthy eating, it has become crucial to produce top-quality tomatoes to fulfill the growing demand. Tomatoes are rich in vitamins, minerals, sugars, dietary fibers, and antioxidants such as vitamin C (VC), phenols, and lycopene, which are helpful to human health [2]. However, tomatoes are among the crops that require the most water. [3].

The scarcity of water resources has gained more attention due to the increase in world population, severe weather conditions, and resource waste [4]. Furthermore, the competition for water use has grown as urbanization and industry continue to advance [5]. Therefore, meeting agriculture’s water needs with available freshwater resources is challenging. They are prompting researchers to focus on finding new water sources, such as saline or brackish waters, which have abundant reserves and are easy to exploit. However, saltwater irrigation provides water to the soil and adds much salt [6]. Thus, many problems occur, including soil salinization, ionic toxicity, physiological drought, and decreased crop production and quality caused by the accumulation of salts in the soil [7,8,9]. Therefore, using saline water scientifically and efficiently has become critical in ensuring drought tolerance and boosting agricultural production. The salinity of irrigation water, frequency, and irrigation rate all affect the increase in soil salinity. Accordingly, many efforts have been made to develop rational irrigation technologies to mitigate the adverse effects of using saltwater irrigation [10,11]. These methods include using water-saving irrigation systems [12] and soil improvement [13] to achieve the goal of reducing the accumulation of salts in the soil [14].

Freshwater shortages in arid and semi-arid regions force farmers to follow deficit irrigation strategies, irrigation with saline water, or their combination. Thus, crops are at risk of drought, salinity, or both [15,16,17]. Meanwhile, the combined effects of salinity stress and drought are more detrimental to the growth and production of vegetable crops than the individual impacts of each stress [15]. Yang et al. [11] state that simultaneous salinity stress and drought reduced tomato fruit yield. Still, mild dehydration at a certain salinity level can aid the osmotic adjustment of the fruit, improving the quality of tomato fruits by increasing the concentration of solutes in the sap of the fruit. Optimal irrigation management and soil improvement are effective methods to confront the adverse effects of abiotic stresses [18].

Biochar has many agricultural, environmental, and economic benefits [19,20]. Adding biochar is an effective way to improve productivity and water use efficiency in the long term. Biochar is a high-carbon, fine-grained organic material produced through pyrolysis, a thermal degradation process of organic matter under temperatures ranging from 300 to 600 °C without oxygen [21,22]. In addition, tomato yield was improved by 55.23% with biochar at a rate of 50 tons ha^−1^ of biochar produced from fruit trees compared to untreated soil [23]. Biochar could enhance soil properties, crop production, and water use efficiency with water scarcity by reducing the adverse impacts of salinity and drought stress [24]. According to Hannachi et al. [25], biochar addition at 4.8 tons/ha enhanced the vegetative growth traits, the number of flowers, and fruit diameter. However, it did not compensate for the yield decrease or reduce the sodium ion content of fruits under salinity stress [26]. Saline water irrigation enhances fruit quality (vitamin C (VC), total acidity (TA), total sugars (TS), and total soluble solids (TSS)). Still, it negatively impacts tomato plant growth and yield, affecting the overall performance of the plants [18]. By changing the soil’s physical properties, applied biochar increases the porosity and water-holding capacity and decreases the bulk density of saline soils [27,28,29]. Adding date palm frond waste biochar reduced bulk density and increased field capacity (FC) in sandy soil compared to untreated soil [30,31].

Biochar addition increased the nutrient content, including N, P, K^+^, and Ca^2+^, in salty soil by reducing nutrient leaching and increasing input [32,33]. Amending sandy soils with biochar might retain more water available for plants and minimize nutrient loss compared to unamended sandy soils [34]. In addition to enhancing the physical properties of the sandy soil, such as decreasing hydraulic conductivity, infiltration rate, and bulk density, adding biochar improves the growth and yield quality of vegetables in saline soil [29,35,36]. Adding the appropriate dose of biochar could increase the yield and enhance tomato fruit quality, including TSS, VC, TA, TS, and lycopene [37,38,39,40]. TSS and VC were slightly increased by adding biochar, but TSS and VC were generally higher with lower irrigation and higher salinity [41]. According to Yang et al. [42], adding biochar led to decreased Na+ uptake and increased K^+^ uptake with all irrigation treatments compared to no treatment. The reduction in Na^+^ content in biochar-treated plants was attributed to the ability of biochar to absorb Na^+^, while the decrease in osmotic pressure was due to the ability of biochar to retain moisture and increase soil mineral content (K^+^, Ca^2+^, and Mg^2+^) with the addition of biochar. As a result, the plant’s uptake of Na^+^ decreased.

Numerous studies have been conducted to highlight the positive impact of biochar on tomato plants when subjected to drought or salinity stress. However, limited research is available on the role of biochar under the combined stress of drought and salinity. Therefore, this study aimed to investigate whether using biochar derived from date palm residues can help mitigate the adverse effects of salinity stress and drought on tomato fruit yield and quality.

## 2. Results and Discussion

### 2.1. The Physical Characteristics and Yield of Tomato Fruits

Used saline water and deficit irrigation significantly reduced fruit physical parameters such as the length and diameter of the fruit, the thickness of the fruit flesh, the fresh and dry average weight of fruit, and the yield. On the other hand, irrigation at 80% of ETc improved the firmness of the fruit’s flesh (Table 1). In contrast, using biochar at a rate of 5% enhanced all fruit physical parameters, fresh and dry weight, and the yield of fruits compared to untreated plants (Table 1). According to Al-Selwey et al. [43] and Sivakumar and Srividhya [44], reducing irrigation water decreased all physical characteristics of tomato fruits (length, diameter, weight, and skin thickness) and yield, as well as saline water irrigation [11,45,46].

Applying biochar positively affected all the fruit’s physical parameters and average fresh and dry weight by irrigating with fresh water at all irrigation levels. Biochar was added to plants and irrigated with saline water, decreasing the fruit’s physical characteristics and fresh and dry weight. This effect was especially noticeable at low irrigation levels of 60% and 40% of ETc. However, irrigation by 80% of ETc, with biochar and irrigating with saline water, improved the fruit flesh’s firmness (Table 2). The decrease in the physical parameters of fruits might be related to the negative impact of water and salt stress on physiological processes within the plant through metabolic imbalances, osmotic stress, nutritional imbalance, and ionic toxicity [22,47]. Applying biochar to soil effectively improves the soil’s physical, chemical, and biological properties, enhancing crop productivity and mitigating the adverse impacts of salinity and drought stresses [48,49].

**Figure 1:**
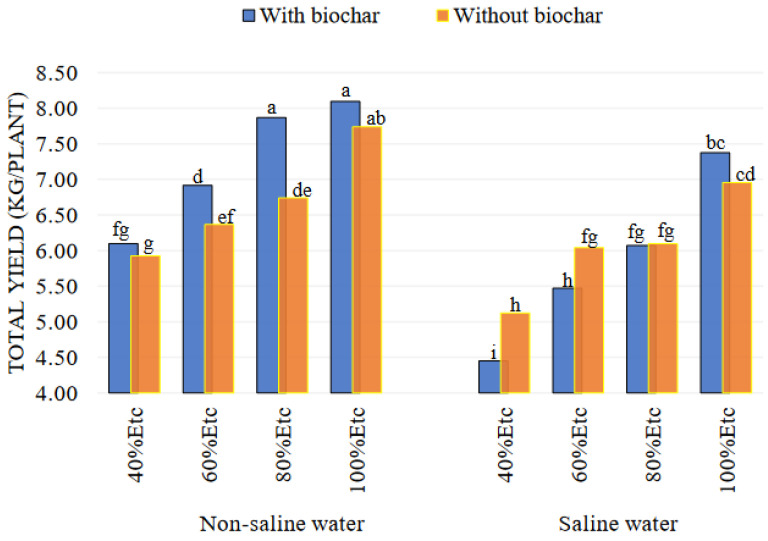
Impacts of interaction between salinity, deficit irrigation levels (ETc), and soil amendments (biochar) on fruit yield (kg/plant). According to the LSD test, columns with the same letter do not show significant differences at the 0.05 probability level.

The yield of tomatoes decreased by 29.33% and 42.51% under the most stressful irrigation levels (60% and 40% of ETc), respectively, when irrigated with saline water and biochar, compared to the control (Figure 1). The negative effect of adding biochar on tomato yield was likely due to the physiological drought caused by the interaction between biochar and deficit irrigation by saline water; this was related to the inability to absorb water by the roots and caused a reduced yield [41]. Additionally, the high pH of the biochar could affect the availability of nutrients for absorption by the plant, resulting in a decrease in tomato production [22,50]. Although adding biochar increased some of the vegetative growth traits, the adverse effects on the yield were not reduced [26], and on the other hand, using freshwater for irrigation, with 5% biochar, enhanced tomato plant productivity under all irrigation levels by 2.97%, 8.67%, 16.74%, and 4.60% for 40, 60, 80, and 100% of ETc, respectively, compared to untreated plants (BC0%) (Figure 1). The increased yield in environments suffering from water stress can be attributed to the ability of biochar to retain water and enhance porosity and nutrients [51,52]. According to Guo et al. [23], applying 50 tons per hectare of the biochar produced from fruit trees increased tomato yield by 55.23% compared to no treatment. Tomato yield increased by 68.57% by adding 20 tons per hectare of biochar [37].

### 2.2. The Qualitative Characteristics

All treatments, such as deficit irrigation, saline water, and the addition of biochar, enhanced the qualitative characteristics of tomato fruits, including vitamin C (VC), total soluble solids (TSS), total titratable acidity (TA), and total sugars (TS) (Table 3). This related water stress enhances the quality of tomato fruits by increasing the concentration of solute in the fruit sap due to a decrease in water flow from the phloem. Our results align with similar research, which indicated that salt and water stress could enhance tomato fruit quality [53,54,55,56,57]. Also, Keabetswe et al. [58] and Zhang et al. [59] corroborated these results and indicated that adding biochar could enhance tomato quality by increasing TSS, TS, TA, and VC.

**Figure 2:**
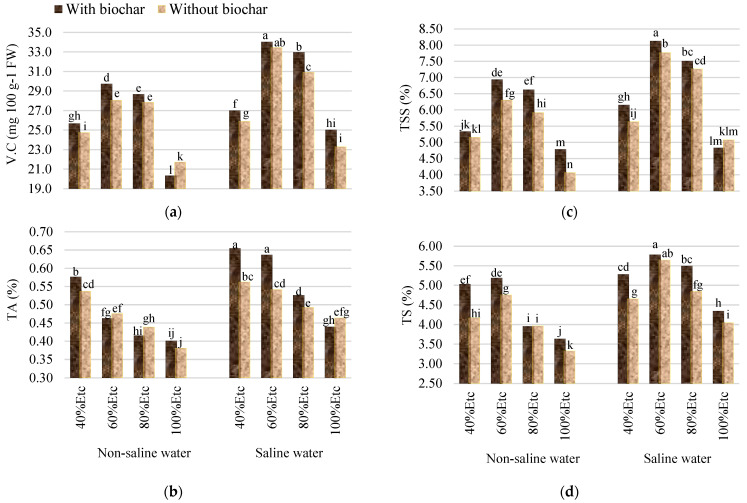
Interaction impacts between salinity (S), irrigation levels (ETc), and soil amendments (biochar) on the quality characteristics of tomato fruits, including vitamin C (V.C.) (**a**), total acidity (TA) (**b**), total soluble solids (TSS) (**c**), and total sugars (TS) (**d**). Columns with the same letter do not show statistically significant differences at the 0.05 probability level, according to the LSD test.

The interaction between salinity, irrigation levels, and biochar led to the improvement in the qualitative characteristics of tomato fruits, as all qualitative characteristics increased with saline water at the irrigation level of 60% of ETc, and added biochar at a rate of 5% was increased by 56.60, 67.19, 99.75, and 73.57% for VC, TA, TSS, and TS, respectively, compared to the control (Figure 2). This improvement in tomato fruit quality under salinity and drought stress can be attributed to increased ion concentrations due to decreased water content in the fruits. Salt and drought stress had similar impacts on fruit quality. According to many studies, water and salt stress could enhance the quality of tomato fruits [60,61,62]. Similar results were obtained by [48]. They found that adding biochar to sandy soil increased the quality parameters of tomato fruits under saline or non-saline irrigation treatments. This can be attributed to the enhancing effects of biochar on soil properties, as evidenced by the availability of nutrients in the soil. Moreover, adding biochar enhanced all qualitative traits of tomato fruits under conditions of irrigation water shortage compared with untreated plants [39,51].

### 2.3. Mineral Content of Tomato Fruits

Deficit irrigation had a negative impact on the nitrogen (N), phosphorus (P), potassium (K^+^), and sodium (Na^+^) content of fruits compared to the control. Thus, irrigation with saline water decreases the fruits’ N, P, and K^+^ content but increases their Na^+^ content. However, added biochar led to decreased Na^+^ and P contents in fruits, before improving N and K^+^ contents (Table 4). Our results agree with those of Ud Din et al. [63] and Atilgan et al. [64], who observed a decrease in the plant uptake of nutrients under the stress of salinity and drought. Researchers have shown that biochar maintains the ion balance and reduces the ion toxicity caused by Na^+^/K^+^ [63]. Due to their structural similarity, the competition between potassium and sodium for their absorption by the plant causes the K^+^ content of the plant to decrease under salt stress [65,66].

**Figure 3:**
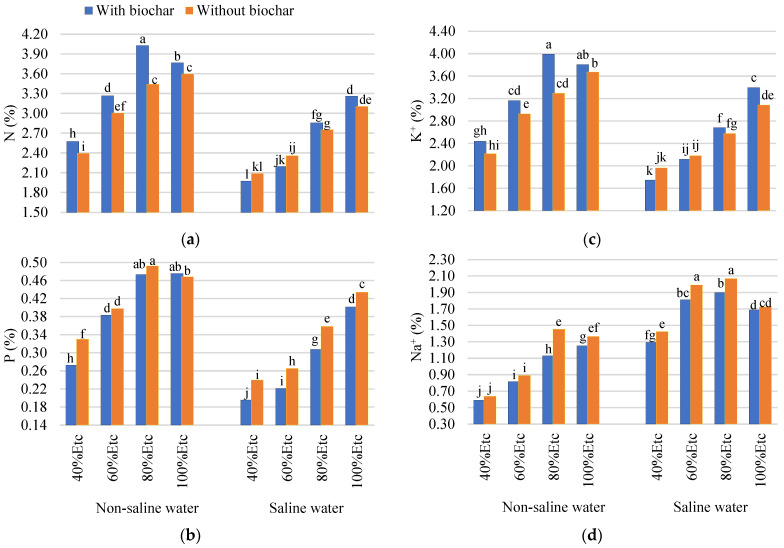
The combined impacts of salinity (S), irrigation levels (ETc), and biochar on the mineral content of tomato fruits. The minerals analyzed were nitrogen (N) (**a**), phosphorus (P) (**b**), potassium (K^+^) (**c**), and sodium (Na^+^) (**d**). The LSD test was used to determine if there were significant differences between the columns. At the 0.05 probability level, no significant difference existed between columns with the same letter.

The interaction between biochar, salinity, and drought stress significantly affected tomato fruits’ mineral content (Figure 3). Biochar addition and saline water irrigation negatively affected the fruits’ N, P, and K+ content, especially under deficit irrigation at 60% and 40% of ETc. Therefore, irrigated by freshwater, with biochar at a rate of 5%, the fruit increased its nitrogen (N) and potassium (K^+^) content with all irrigation treatments. Additionally, the phosphorus (P) content of fruits increased by 100% in ETc compared to untreated plants. Biochar amendments reduced the sodium content of the tomato fruits, which were irrigated by saline and freshwater with all irrigation treatments, compared to no treatment (BC_0%_). The decrease in Na^+^ content with biochar-treated plants may be attributed to (1) the potential of biochar to momentarily adsorb Na^+^, which lowers Na+ concentrations in the soil solution; (2) the low osmotic pressure due to the potential of biochar to be moisture-retaining, thus reducing the concentration of salts (Na^+^); and (3) the increased soil mineral content (K^+^, Ca^2+^, and Mg^2+^) with the addition of biochar. As a result, Na+ was less absorbed by the plant. According to Novak et al. [67], biochar’s high adsorption properties are attributed to its high cation exchange capacity, surface area, and porosity. Biochar might reduce osmotic stress by adsorbing excess Na^+^ from the soil [42]. Similarly, the addition of biochar resulted in a decrease in the uptake of Na+ and an increase in the uptake of K^+^ with all irrigation treatments [25].

## 3. Materials and Methods

### 3.1. Experimental Site

**Figure 4:**
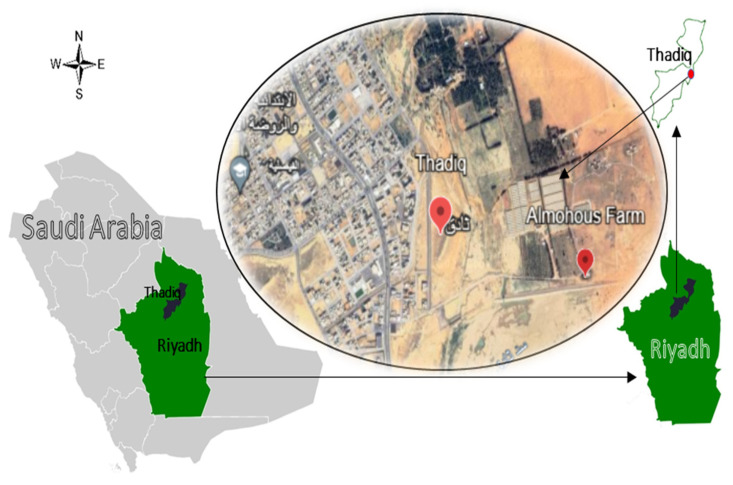
Thadiq Governorate, Riyadh, Saudi Arabia (location of the experiment).

The experiment was conducted in a greenhouse at Al-Mohous Commercial Farm in the Thadiq governorate, 120 km northwest of Riyadh, Saudi Arabia. The farm is situated at latitude 25°17′40″ N and longitude 45°52′55″ E, 722 m above sea level (Figure 4). According to the method explained by Sparks and Page [68], soil samples were taken from the greenhouse before planting for the physical and chemical analysis presented in Table 5, while the chemical analysis of irrigation water shown in Table 5 was carried out according to the method of [69].

### 3.2. Experimental Design

**Figure 5:**
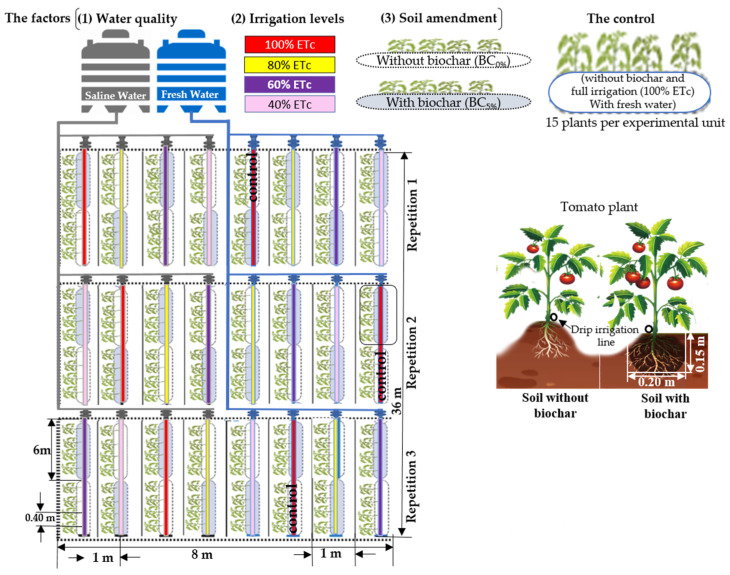
A sketch showing the experimental unit and the random distribution of treatments.

This study comprised sixteen treatments that combined four irrigation levels (40, 60, 80, and 100% of ETc), two treatments for irrigation water quality of 0.9 and 2.3 dS m^−1^ (sodium chloride (NaCl) was used to prepare saline irrigation water), and soil amendments represented by adding 5% of biochar w/w and denoted as (BC_5%_) for treated and (BC_0%_) for untreated soils. The experiment was designed as a randomized complete block (Split-Split-Plot Design) with three replicates. The main plot factor was water quality, irrigation water levels were subplot factors under the main factor, and soil amendments (biochar) were sub-subplot factors. The experiment included 48 experimental units as follows: [2 water quality × 4 irrigation levels × 2 soil amendments (the treated soil (BC_5%_) and the untreated soil (BC_0%_)) × 3 replicates]. The experimental unit included 15 plants and consisted of a line 6 m long and 1 m wide, with a space between 1 m and 0.4 m lines between emitters (2.5 plant m^−2^). The comparison was with full irrigation (100% of ETc) without biochar and salinity (the control) (Figure 5).

The surface drip irrigation system was applied. Irrigation levels were set at 40, 60, 80, and 100% of *ET_c_*, calculated according to [70], depending on the crop coefficient (*K_c_*) values and the daily evapotranspiration amount. To calculate *ET_c_*, the following equation was used to determine *ET_c_*:

(1) $${ET}_{c}=\;E_{o}K_{p}K_{c}$$

where *ET_c_* is crop evapotranspiration (mm), *E_o_* is the evaporation from pan A (mm), *K_P_* is the pan coefficient, and *K_c_* is the crop coefficient.

This study used a commercial hybrid tomato, Tone Guitar. Seeds of the tomato (*Solanum lycopersicum* L.) were sown on 19 September 2021 in foam trays filled with vermiculite and peat moss at a 1:1 ratio (*v/v*) under controlled conditions in a fiberglass greenhouse. After four weeks, seedlings were transferred to a control greenhouse. The average greenhouse temperature and relative air humidity were 24 ± 1.5 °C and 75 ± 2% during the growth stages. General agricultural recommendations were followed for commercial tomato production, like soil sterilization, pest control, and fertilization, with fertilizers applied at a rate recommended by local farmers of 285, 142, and 238 kg per hectare (N, P_2_O_5_, and K_2_O), respectively.

### 3.3. Biochar Preparation

**Figure 6:**
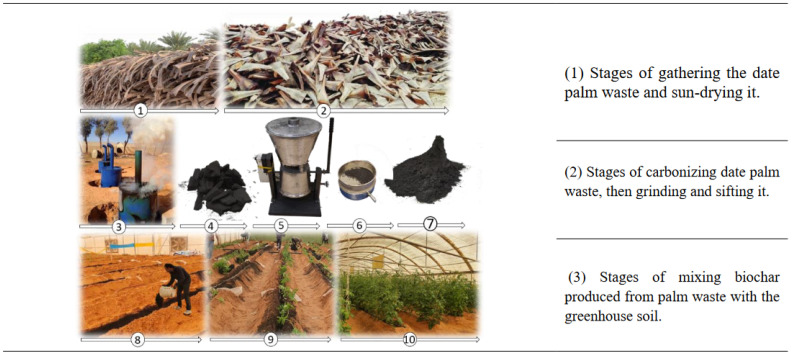
Stages of preparing biochar and mixing it with greenhouse soil.

In this study, the preparation of biochar used the waste of date palm fronds, collected and dried, cut into small pieces, and then placed into a kiln with minimal oxygen and pyrolyzed at a temperature of 450 °C ± 10 °C. After completion of the carbonization process, further details can be found in [71]. The biochar was ground using an electric grinder and sieved through a 2 mm sieve. They were finally mixed with the soil, as shown in Figure 6. The surface area was determined using the Micromeritics ASAP 2020 BET. A pH meter and conductivity meter were used to measure the pH and EC of an aqueous extract made from biochar 1:10 (*w/v*). A CHN analyzer (Series II; Perkin Elmer, Waltham, MA, USA) was used to measure the contents of carbon (C), hydrogen (H), and nitrogen (N). According to the American Society for Testing and Materials, the biochar’s ash, mobile materials, fixed carbon, and moisture content were calculated [72]. The chemical and physical properties of the biochar are shown in Table 5.

### 3.4. Physicochemical Properties of Tomato Fruits

At the peak of the harvesting period, ten perfectly red-ripe tomato fruits were collected randomly per experimental unit. Fruit quality indicators were measured using a vernier caliper, including fruit length, diameter, average fresh and dry weight of fruit (single fruit weight), and thickness and firmness of the fruit flesh. Flesh firmness was measured using a firmness tester (FT 40, Wagner Instruments, Greenwich, CT, USA). A digital weighing balance determined the fruit’s fresh and dry weights. The fresh weight was measured directly, and the dry weight was measured after the fruits dried in a forced-air oven until the weight remained constant. Qualitative characteristics were measured according to the method explained in [73]. The fruits were rinsed once with fresh water and twice with distilled water and sliced. Afterward, the slices were mixed using an electric blender to extract the juice to measure the following: total soluble solids (TSS), vitamin C, total titratable acidity (TA), and total sugars (TS). A portable digital refractometer (PR-101 model, ATAGO, Tokyo, Japan) was used to measure the TSS content of tomato fruit juice. VC was determined by titration using the 2 and 6 dichlorophenolindophenol dyes. TA was estimated using potentiometric titration with 0.1 M NaOH up to pH 8.1 using the phenolphthalein indicator and is expressed as the percentage of citric acid in the fruit juice. Using a digital weighing balance, the weight of all fruits was recorded during the fruiting stage until the end of harvest to estimate the total yield (kg plant^−1^).

To analyze the nutrients (N, P, K, and Ca), 100 g of the homogenized tomato sample was dried in a drying oven and crushed using an electric grinder. After that, 10 mL of concentrated sulfuric acid (H_2_SO_4_) was added to 0.2 g of the sample in a glass tube. The tubes were placed on a block digester until they turned transparent, and the sample was supplied with distilled water to the final volume (100 mL) [74]. The nitrogen (N) content of the digested samples was determined using the Kjeldahl digestion method described by [75] using the Kjeldahl device (DF-4S Mitamura Riken Kogyo Inc., Tokyo, Japan). According to Kacar and Inal [76], phosphorus (P) content was determined by vanadate-molybdate colorimetry using a spectrophotometer (UV/Vis Model-9100, LabTech, Littleton, MA, USA). Potassium (K^+^) and sodium (Na^+^) contents were determined using the flame photometer method (Model 1382/1385 S/No. 1403149, London, UK) as described by [77].

### 3.5. Data Analysis

The Statistical Package for the Social Sciences (SPSS) program was used to compare the means [78]. Analysis of variance (ANOVA) was used to compare the data statistically, and the least significant difference (LSD) test was performed at a significance level of 0.05, according to [79].

## 4. Conclusions

Improving the productivity and quality of tomato crops has become increasingly important, especially in arid regions with sandy soil and low-quality irrigation water. Enhancing the quality of sandy soil is necessary to mitigate the negative consequences of salinity and drought stress. Our study used 5% biochar to improve sandy soil under salinity and drought stress. The results of this study confirmed that salinity and drought stress have a negative impact on the yield of tomatoes and the physical and chemical properties of the fruits. However, applying 5% biochar significantly enhanced the yield and all fruit characteristics compared to the fruits of untreated plants. The combination of salinity and biochar had a negative impact on the physical properties and mineral content of fruits. As a result, the yield decreased by 42.51% and 29.33%, with deficit irrigation levels at 40 and 60% of ETc, respectively, compared to the control. However, when biochar was combined with saline water at 60% of ETc, the flesh firmness and quality characteristics of tomato fruits (VC, TA, TSS, and TS) were improved. Additionally, adding biochar to the plants reduced the sodium content of the fruits under all irrigation levels compared to untreated plants (BC_0%_). Overall, using biochar with saline water (S 2.3 ds m^−1^) and deficit irrigation (60 and 80% of ETc) could improve the quality characteristics of tomato fruits, but at the same time, the yield decreased.

## Figures and Tables

**Table 1:** The effect of salinity (S), irrigation levels, and biochar (BC) on fruit physical parameters (length and diameter of the fruit and the thickness and firmness of the fruit’s flesh).

Treatments	Fruit Length (mm)	Fruit Diameter (mm)	Fruit Flesh Thickness (mm)	Fruit Firmness (N)	FFW (g)	FDW (g)	Yield kg/Plant
Salinity							
S _0.9 ds m_^−1^	63.34 a	71.89 a	8.50 a	6.17 a	175.69 a	9.46 a	6.97 a
S _2.3 ds m_^−1^	58.55 b	63.48 b	6.56 b	6.14 a	132.54 b	7.95 b	5.95 b
Irrigation Levels (% of ETc)							
100	68.59 a	77.08 a	9.51 a	6.60 b	189.37 a	9.73 a	7.54 a
80	63.46 b	71.90 b	8.21 b	7.68 a	176.04 b	9.48 b	6.69 b
60	58.66 c	63.90 c	6.76 c	5.61 c	140.12 c	8.25 c	6.20 c
40	53.08 d	57.86 d	5.64 d	4.73 d	110.92 d	7.36 d	5.40 d
Biochar							
BC_0%_	58.98 b	66.62 b	7.34 b	5.90 b	151.10 b	8.60 b	6.37 b
BC_5%_	62.91 a	68.75 a	7.27 a	6.41 a	157.13 a	8.82 a	6.54 a

The LSD test, at the 0.05 probability level, indicates that there is no significant difference between values that have the same letter.

**Table 2:** Impacts of interaction between salinity (S), irrigation levels, and biochar (BC) on the physical characteristics of fruits (length and diameter of the fruit and the thickness and firmness of the fruit’s flesh), fruit fresh weight (FFW), and fruit dry weight (FDW).

Salinity	Irrigation Levels (% of ETc)	Biochar (%)	Fruit Length (mm)	Fruit Diameter (mm)	Fruit Flesh Thickness (mm)	Fruit Firmness (N)	FFW (g)	FDW (g)
S _0.9 ds m_^−1^	100	BC_0%_	66.34 c	78.54 bc	9.76 b	5.97 fg	198.83 bc	9.60 de
		BC_5%_	74.91 a	81.44 a	10.73 a	6.64 d	212.82 a	10.16 a–c
	80	BC_0%_	61.37 de	73.47 d	8.56 cd	7.01 c	192.14 c	10.24 ab
		BC_5%_	72.41 a	80.61 ab	10.25 ab	7.94 a	206.84 ab	10.39 a
	60	BC_0%_	60.60 e	66.56 f	7.63 e	5.73 gh	155.40 f	8.73 f
		BC_5%_	62.12 de	70.01 e	8.25 d	6.12 ef	173.83 de	9.75 cd
	40	BC_0%_	52.26 g	60.47 h	6.09 g	4.71 k	123.92 h	8.13 g
		BC_5%_	56.70 f	64.01 g	6.75 f	5.26 ij	141.74 g	8.67 f
S _2.3 ds m_^−1^	100	BC_0%_	63.99 cd	72.12 de	8.49 d	6.36 de	168.36 e	9.23 e
		BC_5%_	69.11 b	76.23 c	9.06 c	7.44 b	177.45 d	9.95 b–d
	80	BC_0%_	60.49 e	66.26 f	7.19 of	7.73 ab	154.97 f	8.60 f
		BC_5%_	59.55 e	67.26 fg	6.86 f	8.05 a	150.23 f	8.71 f
	60	BC_0%_	55.95 f	60.40 h	5.86 g	5.10 j	121.09 h	7.60 h
		BC_5%_	55.99 f	58.64 h	5.30 h	5.50 hi	110.17 i	6.90 i
	40	BC_0%_	50.88 g	55.17 i	5.14 h	4.60 kl	94.09 j	6.63 i
		BC_5%_	52.46 g	51.79 j	4.58 i	4.34 l	83.94 k	6.00 j

At the 0.05 probability level, the LSD test showed that there was no significant difference between values that have the same letter.

**Table 3:** The effect of salinity, irrigation water levels, and soil amendments (biochar) on the qualitative characteristics of tomato fruits, including total soluble solids (TSS), vitamin C (V.C.), total titratable acidity (TA), and total sugars (TS).

Treatments	V.C (mg 100 g^−1^ FW)	TA (%)	TSS (%)	TS (%)
Salinity				
S _0.9 ds m_^−1^	25.85 b	0.461 b	5.64 b	4.25 b
S _2.3 ds m_^−1^	29.08 a	0.540 a	6.55 a	5.01 a
Irrigation Levels (% of ETc)				
100	22.61 d	0.422 d	4.69 d	3.84 d
80	30.10 b	0.468 c	6.83 b	4.57 c
60	31.32 a	0.529 b	7.29 a	5.34 a
40	25.84 c	0.583 a	5.57 c	4.78 b
Biochar				
BC_0%_	27.00 b	0.487 b	5.90 b	4.43 b
BC_5%_	27.93 a	0.515 a	6.28 a	4.84 a

At the 0.05 probability level, the LSD test showed that there was no significant difference between values that have the same letter.

**Table 4:** Impact of salinity, irrigation water level, and biochar on the mineral content of tomato fruits, such as nitrogen (N), phosphorus (P), potassium (K^+^), and sodium (Na^+^).

Treatments	N (%)	P (%)	K (%)	Na^+^ (%)
Salinity				
S _0.9 ds m_^−1^	3.26 a	0.412 a	3.19 a	1.02 b
S _2.3 ds m_^−1^	2.57 b	0.303 b	2.47 b	1.74 a
Irrigation Levels (%ETc)				
100	3.43 a	0.445 a	3.49 a	1.51 b
80	3.27 b	0.408 b	3.14 b	1.64 a
60	2.71 c	0.317 c	2.60 c	1.38 c
40	2.26 d	0.260 d	2.09 d	0.99 d
Biochar				
BC_0%_	2.84 b	0.373 a	2.74 b	1.44 a
BC_5%_	2.99 a	0.341 b	2.92 a	1.31 b

According to the LSD test, values that have the same letter do not significantly differ at the 0.05 probability level.

**Table 5:** Chemical properties of irrigation water and physicochemical properties of biochar and soil.

Parameters	Moisture (%)	Resident Material (%)	SA m^2^ g^−1^	OM %	Ash %	pH	EC dS m^−1^	N %	P %	K %	C %	H %	Na %	C/N Ratio
biochar	3.53	47.90	237.80	30.33	25.70	8.82	3.71	0.24	0.22	0.88	60	3.44	5.63	250:1
								Cations (meql^−1^)				Anions (meql^−1^)		
Irrigation Water								Ca^2+^	Mg^2+^	K^+^	Na^+^	CO_3_^2−^	Cl^−^	HCO_3_^−^
		Fresh			2.78	7.21	0.93	3.19	2.54	0.13	4.70	0.00	7.93	2.32
		Saline			13.32	7.52	2.30	2.80	2.20	0.29	21.04	0.00	21.29	2.86
Chemical properties of soil					2.02	7.27	2.46	10.92	2.25	5.10	3.80	0.00	4.50	18.3
Soil Type			Sand (%)			Silt (%)			Clay (%)			Soil texture		
			80			13			7			Loamy sand		

SA = surface area, OM = organic matter.

## Data Availability

Data are available at the request of the authors.

## References

[1] Nikolaou G., Neocleous D., Christou A., Polycarpou P., Kitta E., Katsoulas N. (2021). Energy and Water Related Parameters in Tomato and Cucumber Greenhouse Crops in Semiarid Mediterranean Regions. A Review, Part I: Increasing Energy Efficiency. Horticulturae.

[2] Abdul-Hammed M., Bolarinwa I.F., Adebayo L.O., Akindele S.L. (2016). Kinetics of the degradation of carotenoid antioxidants in tomato paste. Adv. J. Food Sci. Technol..

[3] Patanè C., Tringali S., Sortino O. (2011). Effects of deficit irrigation on biomass, yield, water productivity and fruit quality of processing tomato under semi-arid Mediterranean climate conditions. Sci. Hortic..

[4] Gao Y., Shao G., Lu J., Zhang K., Wu S., Wang Z. (2020). Effects of biochar application on crop water use efficiency depend on experimental conditions: A meta-analysis. Field Crops Res..

[5] Du Y.-D., Niu W.-Q., Gu X.-B., Zhang Q., Cui B.-J., Zhao Y. (2018). Crop yield and water use efficiency under aerated irrigation: A meta-analysis. Agric. Water Manag..

[6] Bañón D., Lorente B., Ortuño M.F., Bañón S., Sánchez-Blanco M.J., Alarcón J.J. (2022). Effects of saline irrigation on the physiology and ornamental quality of Euphorbia Ascot Rainbow and its relationship with salinity indexes based on the bulk electrical conductivity. Sci. Hortic..

[7] Su H., Sun H., Dong X., Chen P., Zhang X., Tian L., Liu X., Wang J. (2021). Did manure improve saline water irrigation threshold of winter wheat? A 3-year field investigation. Agric. Water Manag..

[8] Izadi Y., Moosavi S.A., Gharineh M.H. (2022). Salinity affects eco-physiological aspects and biochemical compositions in chia (*Salvia hispanica* L.) during germination and seedling growth. Sci. Hortic..

[9] Loganathachetti D.S., Alhashmi F., Chandran S., Mundra S. (2022). Irrigation water salinity structures the bacterial communities of date palm (*Phoenix dactylifera*)-associated bulk soil. Front. Plant Sci..

[10] Li J., Gao Y., Zhang X., Tian P., Li J., Tian Y. (2019). Comprehensive comparison of different saline water irrigation strategies for tomato production: Soil properties, plant growth, fruit yield and fruit quality. Agric. Water Manag..

[11] Li J., Chen J., He P., Chen D., Dai X., Jin Q., Su X. (2022). The optimal irrigation water salinity and salt component for high-yield and good-quality of tomato in Ningxia. Agric. Water Manag..

[12] Li X., Kang Y., Wan S., Chen X., Xu J. (2015). Effect of drip-irrigation with saline water on Chinese rose (*Rosa chinensis*) during reclamation of very heavy coastal saline soil in a field trial. Sci. Hortic..

[13] Song J., Zhang H., Chang F., Yu R., Wang J., Wang X., Li Y. (2022). If the combination of straw interlayer and irrigation water reduction maintained sunflower yield by boosting soil fertility and improving bacterial community in arid and saline areas. Agric. Water Manag..

[14] Han X., Kang Y., Wan S., Li X. (2022). Effect of salinity on oleic sunflower (*Helianthus annuus* Linn.) under drip irrigation in arid area of Northwest China. Agric. Water Manag..

[15] Ors S., Suarez D.L. (2017). Spinach biomass yield and physiological response to interactive salinity and water stress. Agric. Water Manag..

[16] Sahin U., Ekinci M., Ors S., Turan M., Yildiz S., Yildirim E. (2018). Effects of individual and combined effects of salinity and drought on physiological, nutritional and biochemical properties of cabbage (*Brassica oleracea* var. capitata). Sci. Hortic..

[17] Caliskan O., Radusiene J., Temizel K.E., Staunis Z., Cirak C., Kurt D., Odabas M.S. (2017). The effects of salt and drought stress on phenolic accumulation in greenhouse-grown Hypericum pruinatum. Ital. J. Agron..

[18] Gao Y., Shao G., Cui J., Lu J., Tian L., Song E., Zeng Z. (2023). Effects of Drought Hardening and Saline Water Irrigation on the Growth, Yield, and Quality of Tomato. Agronomy.

[19] Obadi A., AlHarbi A., Abdel-Razzak H., Al-Omran A. (2020). Biochar and compost as soil amendments: Effect on sweet pepper (*Capsicum annuum* L.) growth under partial root zone drying irrigation. Arab. J. Geosci..

[20] Oni B.A., Oziegbe O., Olawole O.O. (2019). Significance of biochar application to the environment and economy. Ann. Agric. Sci..

[21] Lehmann J., Joseph S. (2015). Biochar for Environmental Management: Science, Technology and Implementation.

[22] Obadi A., Alharbi A., Alomran A., Alghamdi A.G., Louki I., Alkhasha A. (2023). Effect of Biochar Application on Morpho-Physiological Traits, Yield, and Water Use Efficiency of Tomato Crop under Water Quality and Drought Stress. Plants.

[23] Guo L., Yu H., Kharbach M., Zhang W., Wang J., Niu W. (2021). Biochar improves soil-tomato plant, tomato production, and economic benefits under reduced nitrogen application in northwestern China. Plants.

[24] Faloye O., Alatise M., Ajayi A., Ewulo B. (2019). Effects of biochar and inorganic fertiliser applications on growth, yield and water use efficiency of maize under deficit irrigation. Agric. Water Manag..

[25] Hannachi S., Signore A., Mechi L. (2023). Alleviation of Associated Drought and Salinity Stress’ Detrimental Impacts on an Eggplant Cultivar (‘Bonica F1’) by Adding Biochar. Plants.

[26] Hazman M.Y., El-Sayed M.E., Kabil F.F., Helmy N.A., Almas L., McFarland M., Shams El Din A., Burian S. (2022). Effect of biochar application to fertile soil on tomato crop production under Saline irrigation regime. Agronomy.

[27] Chaganti V.N., Crohn D.M. (2015). Evaluating the relative contribution of physiochemical and biological factors in ameliorating a saline–sodic soil amended with composts and biochar and leached with reclaimed water. Geoderma.

[28] Obia A., Mulder J., Martinsen V., Cornelissen G., Børresen T. (2016). In situ effects of biochar on aggregation, water retention and porosity in light-textured tropical soils. Soil Tillage Res..

[29] Liu Q., Meki K., Zheng H., Yuan Y., Shao M., Luo X., Li X., Jiang Z., Li F., Xing B. (2023). Biochar application in remediating salt-affected soil to achieve carbon neutrality and abate climate change. Biochar.

[30] Alghamdi A.G., Aly A.A., Al-Omran A.M., Louki I.I., Alkhasha A. (2023). Tomato Yield Responses to Deficit Irrigation and Partial Root Zone Drying Methods Using Biochar: A Greenhouse Experiment in a Loamy Sand Soil Using Fresh and Saline Irrigation Water. Water.

[31] Al-Omran A.M., Mousa M.A., Alghamdi A.G., Alkhasha A. (2023). Impact of Nanoparticles from Ball-Milled Date Palm Biochar on the Hydro-Physical Characteristics of Sandy Soils. Appl. Sci..

[32] Drake J.A., Cavagnaro T.R., Cunningham S.C., Jackson W.R., Patti A.F. (2016). Does biochar improve establishment of tree seedlings in saline sodic soils?. Land Degrad. Dev..

[33] Kanthle A.K., Lenka N.K., Lenka S., Tedia K. (2016). Biochar impact on nitrate leaching as influenced by native soil organic carbon in an Inceptisol of central India. Soil Tillage Res..

[34] Dokoohaki H., Miguez F.E., Laird D., Horton R., Basso A.S. (2017). Assessing the biochar effects on selected physical properties of a sandy soil: An analytical approach. Commun. Soil Sci. Plant Anal..

[35] Dahlawi S., Naeem A., Rengel Z., Naidu R. (2018). Biochar application for the remediation of salt-affected soils: Challenges and opportunities. Sci. Total Environ..

[36] Zheng H., Wang X., Chen L., Wang Z., Xia Y., Zhang Y., Wang H., Luo X., Xing B. (2018). Enhanced growth of halophyte plants in biochar-amended coastal soil: Roles of nutrient availability and rhizosphere microbial modulation. Plant Cell Environ..

[37] Zeeshan M., Ahmad W., Hussain F., Ahamd W., Numan M., Shah M., Ahmad I. (2020). Phytostabalization of the heavy metals in the soil with biochar applications, the impact on chlorophyll, carotene, soil fertility and tomato crop yield. J. Clean. Prod..

[38] Guo L., Yu H., Niu W., Kharbach M. (2021). Biochar promotes nitrogen transformation and tomato yield by regulating nitrogen-related microorganisms in tomato cultivation soil. Agronomy.

[39] Akhtar S.S., Li G., Andersen M.N., Liu F. (2014). Biochar enhances yield and quality of tomato under reduced irrigation. Agric. Water Manag..

[40] Almaroai Y.A., Eissa M.A. (2020). Effect of biochar on yield and quality of tomato grown on a metal-contaminated soil. Sci. Hortic..

[41] Wu Z., Fan Y., Qiu Y., Hao X., Li S., Kang S. (2022). Response of yield and quality of greenhouse tomatoes to water and salt stresses and biochar addition in Northwest China. Agric. Water Manag..

[42] Yang A., Akhtar S.S., Li L., Fu Q., Li Q., Naeem M.A., He X., Zhang Z., Jacobsen S.-E. (2020). Biochar mitigates combined effects of drought and salinity stress in quinoa. Agronomy.

[43] Al-Selwey W., Alsadon A., Al-Doss A., Solieman T., Dewir Y., Ibrahim A. (2021). Effect of deficit irrigation on total yield, fruit physical characteristics and nutritional value in four drought tolerant tomato (*Solanum lycopersicum* L.) genotypes. J. Agric. Sci. Technol..

[44] Sivakumar R., Srividhya S. (2016). Impact of drought on flowering, yield and quality parameters in diverse genotypes of tomato (*Solanum lycopersicum* L.). Adv. Hortic. Sci..

[45] Farooq H., Bashir M.A., Khalofah A., Khan K.A., Ramzan M., Hussain A., Wu L., Simunek L., Aziz I., Samdani M.S. (2021). Interactive effects of saline water irrigation and nitrogen fertilization on tomato growth and yield. Fresenius Environ. Bull..

[46] Al-Harbi A.R., Al-Omran A.M., Alenazi M.M., Wahb-Allah M.A. (2015). Salinity and Deficit Irrigation Influence Tomato Growth, Yield and Water Use Efficiency at Different Developmental Stages. Int. J. Agric. Biol..

[47] Angon P.B., Tahjib-Ul-Arif M., Samin S.I., Habiba U., Hossain M.A., Brestic M. (2022). How do plants respond to combined drought and salinity stress?—A systematic review. Plants.

[48] Usman A.R.A., Al-Wabel M.I., Abdulaziz A.-H., Mahmoud W.-A., El-Naggar A.H., Ahmad M., Abdulelah A.-F., Abdulrasoul A.-O. (2016). Conocarpus biochar induces changes in soil nutrient availability and tomato growth under saline irrigation. Pedosphere.

[49] Zhang W., Wei J., Guo L., Fang H., Liu X., Liang K., Niu W., Liu F., Siddique K.H. (2023). Effects of two biochar types on mitigating drought and salt stress in tomato seedlings. Agronomy.

[50] Blok C., Van der Salm C., Hofland-Zijlstra J., Streminska M., Eveleens B., Regelink I., Fryda L., Visser R. (2017). Biochar for horticultural rooting media improvement: Evaluation of biochar from gasification and slow pyrolysis. Agronomy.

[51] Agbna G.H., Dongli S., Zhipeng L., Elshaikh N.A., Guangcheng S., Timm L.C. (2017). Effects of deficit irrigation and biochar addition on the growth, yield, and quality of tomato. Sci. Hortic..

[52] Hou J., Zhang J., Liu X., Ma Y., Wei Z., Wan H., Liu F. (2023). Effect of biochar addition and reduced irrigation regimes on growth, physiology and water use efficiency of cotton plants under salt stress. Ind. Crops Prod..

[53] Colimba-Limaico J.E., Zubelzu-Minguez S., Rodríguez-Sinobas L. (2022). Optimal Irrigation Scheduling for Greenhouse Tomato Crop (*Solanum Lycopersicum* L.) in Ecuador. Agronomy.

[54] Liu H., Li H., Ning H., Zhang X., Li S., Pang J., Wang G., Sun J. (2019). Optimizing irrigation frequency and amount to balance yield, fruit quality and water use efficiency of greenhouse tomato. Agric. Water Manag..

[55] Lipan L., Issa-Issa H., Moriana A., Zurita N.M., Galindo A., Martín-Palomo M.J., Andreu L., Carbonell-Barrachina Á.A., Hernández F., Corell M. (2021). Scheduling regulated deficit irrigation with leaf water potential of cherry tomato in greenhouse and its effect on fruit quality. Agriculture.

[56] Zhang X., Tang H., Du H., Bao Z., Shi Q. (2020). Sugar metabolic and N-glycosylated profiles unveil the regulatory mechanism of tomato quality under salt stress. Environ. Exp. Bot..

[57] Alshami A.K., El-Shafei A., Al-Omran A.M., Alghamdi A.G., Louki I., Alkhasha A. (2023). Responses of Tomato Crop and Water Productivity to Deficit Irrigation Strategies and Salinity Stress in Greenhouse. Agronomy.

[58] Keabetswe L., Shao G.C., Cui J., Lu J., Stimela T. (2019). A combination of biochar and regulated deficit irrigation improves tomato fruit quality: A comprehensive quality analysis. Folia Hortic..

[59] Zhang C., Li X., Yan H., Ullah I., Zuo Z., Li L., Yu J. (2020). Effects of irrigation quantity and biochar on soil physical properties, growth characteristics, yield and quality of greenhouse tomato. Agric. Water Manag..

[60] Yang H., Du T., Mao X., Ding R., Shukla M.K. (2019). A comprehensive method of evaluating the impact of drought and salt stress on tomato growth and fruit quality based on EPIC growth model. Agric. Water Manag..

[61] Wu Y., Yan S., Fan J., Zhang F., Xiang Y., Zheng J., Guo J. (2021). Responses of growth, fruit yield, quality and water productivity of greenhouse tomato to deficit drip irrigation. Sci. Hortic..

[62] Mitchell J., Shennan C., Grattan S., May D. (1991). Tomato fruit yields and quality under water deficit and salinity. J. Am. Soc. Hortic. Sci..

[63] Ud Din M.M., Khan M.I., Azam M., Ali M.H., Qadri R., Naveed M., Nasir A. (2023). Effect of biochar and compost addition on mitigating salinity stress and improving fruit quality of tomato. Agronomy.

[64] Atilgan A., Rolbiecki R., Saltuk B., Jagosz B., Arslan F., Erdal I., Aktas H. (2022). Deficit Irrigation Stabilizes Fruit Yield and Alters Leaf Macro and Micronutrient Concentration in Tomato Cultivation in Greenhouses: A Case Study in Turkey. Agronomy.

[65] Balasubramaniam T., Shen G., Esmaeili N., Zhang H. (2023). Plants’ Response Mechanisms to Salinity Stress. Plants.

[66] Zhang M., Liang X., Wang L., Cao Y., Song W., Shi J., Lai J., Jiang C. (2019). A HAK family Na+ transporter confers natural variation of salt tolerance in maize. Nat. Plants.

[67] Novak J.M., Busscher W.J., Watts D.W., Amonette J.E., Ippolito J.A., Lima I.M., Gaskin J., Das K., Steiner C., Ahmedna M. (2012). Biochars impact on soil-moisture storage in an ultisol and two aridisols. Soil Sci..

[68] Sparks D.L., Page A.L., Helmke P.A., Loeppert R.H. (2020). Methods of Soil Analysis, Part 3: Chemical Methods.

[69] Maiti S. (2004). Handbook of Methods in Environmental Studies, 1: Water and Wastewater Analysis.

[70] Allen R.G., Pereira L.S., Raes D., Smith M. (1998). Crop Evapotranspiration-Guidelines for Computing Crop Water Requirements-FAO Irrigation and Drainage Paper 56.

[71] Usman A.R., Abduljabbar A., Vithanage M., Ok Y.S., Ahmad M., Ahmad M., Elfaki J., Abdulazeem S.S., Al-Wabel M.I. (2015). Biochar production from date palm waste: Charring temperature induced changes in composition and surface chemistry. J. Anal. Appl. Pyrolysis.

[72] (1989). Standard Methods for Chemical Analysis of Wood Charcoal.

[73] AOAC—Association of Official Agricultural Chemists (2000). Official Method of Analysis.

[74] Estefan G. (2013). Methods of Soil, Plant, and Water Analysis: A Manual for the West Asia and North Africa Region.

[75] Davidson J., Mathieson J., Boyne A. (1970). The use of automation in determining nitrogen by the Kjeldahl method, with final calculations by computer. Analyst.

[76] Kacar B., Inal A. (2008). Plant analysis. Nobel publication no: 1241. Appl. Sci..

[77] Chapman H., Pratt P. (1961). Methods of Analysis for Soils, Plants and Waters.

[78] IBM Corp Released 2019. IBM SPSS Statistics for Windows.

[79] Steel R.G.D., Torrie J.H. (1960). Principles and Procedures of Statistics.

